# Relaxin Inhibits Ventricular Arrhythmia and Asystole in Rats With Pulmonary Arterial Hypertension

**DOI:** 10.3389/fcvm.2021.668222

**Published:** 2021-07-06

**Authors:** Brian Martin, Rebecca R. Vanderpool, Brian L. Henry, Joshua B. Palma, Beth Gabris, Yen-Chun Lai, Jian Hu, Stevan P. Tofovic, Rajiv P. Reddy, Ana L. Mora, Mark T. Gladwin, Guillermo Romero, Guy Salama

**Affiliations:** ^1^Department of Medicine, University of Pittsburgh, Pittsburgh, PA, United States; ^2^Heart and Vascular Institute, University of Pittsburgh, Pittsburgh, PA, United States; ^3^Department of Bioengineering, University of Pittsburgh, Pittsburgh, PA, United States; ^4^Vascular Medicine Institute, University of Pittsburgh, Pittsburgh, PA, United States; ^5^Department of Pharmacology and Chemical Biology, University of Pittsburgh, Pittsburgh, PA, United States; ^6^Aging Institute, University of Pittsburgh, Pittsburgh, PA, United States

**Keywords:** arrhythmia, sudden cardiac death, fibrosis, metabolism, cardiovascular disease, relaxin, cardiac arrest, thiol oxidation-reduction

## Abstract

Pulmonary arterial hypertension (PAH) leads to right ventricular cardiomyopathy and cardiac dysfunctions where in the clinical setting, cardiac arrest is the likely cause of death, in ~70% of PAH patients. We investigated the cardiac phenotype of PAH hearts and tested the hypothesis that the insulin-like hormone, Relaxin could prevent maladaptive cardiac remodeling and protect against cardiac dysfunctions in a PAH animal model. PAH was induced in rats with sugen (20 mg/kg), hypoxia then normoxia (3-weeks/each); relaxin (RLX = 0, 30 or 400 μg/kg/day, *n* ≥ 6/group) was delivered subcutaneously (6-weeks) with implanted osmotic mini-pumps. Right ventricle (RV) hemodynamics and Doppler-flow measurements were followed by cardiac isolation, optical mapping, and arrhythmia phenotype. Sugen-hypoxia (SuHx) treated rats developed PAH characterized by higher RV systolic pressures (50 ± 19 vs. 22 ± 5 mmHg), hypertrophy, reduced stroke volume, ventricular fibrillation (VF) (*n* = 6/11) and bradycardia/arrest (*n* = 5/11); both cardiac phenotypes were suppressed with dithiothreitol (DTT = 1 mM) (*n* = 0/2/group) or RLX (low or high dose, *n* = 0/6/group). PAH hearts developed increased fibrosis that was reversed by RLX-HD, but not RLX-LD. Relaxin decreased Nrf2 and glutathione transferases but not glutathione-reductase. High-dose RLX improved pulmonary arterial compliance (measured by Doppler flow), suppressed VF even after burst-pacing, *n* = 2/6). Relaxin suppressed VF and asystole through electrical remodeling and by reversing thiol oxidative stress. For the first time, we showed two cardiac phenotypes in PAH animals and their prevention by RLX. Relaxin may modulate maladaptive cardiac remodeling in PAH and protect against arrhythmia and cardiac arrest.

## Introduction

Pulmonary arterial hypertension (PAH) is a progressive disease of the pulmonary vasculature that consists of endothelial dysfunction, increased pulmonary artery (PA) contractility, and proliferation and remodeling of endothelial and smooth muscle cells (1). Although cardiac arrhythmias play an important role in the morbidity and mortality of PAH patients (2), little is known about PAH arrhythmia phenotypes. In PAH-patients, right-sided cardiac remodeling in response to chronic pressure and volume overload sets the stage for an arrhythmogenic substrate. We investigated the cardiac electrophysiology of an established model of PAH, the sugen-hypoxia (SuHx) rat model of PAH which creates sustained severe pulmonary hypertension and many features of pulmonary arteriopathy seen in human PAH (3, 4). Although this model recapitulates the histological and hemodynamic effects of PAH, it has not been characterized from an electrophysiological perspective.

Relaxin (RLX), an insulin-like hormone, was first characterized for its role in reproduction and then found to have marked cardiovascular protective actions (5). In pre-clinical models of pulmonary hypertension, RLX was found to reverse pulmonary fibrosis and alveolar thickening (6, 7). RLX deficient mice developed age-dependent progressive lung fibrosis associated with increased tissue weight, collagen content, and alveolar congestion, which were reversed with exogenous RLX (8). Relaxin also increased arterial compliance, cardiac output, and renal blood flow (9–11). We showed that RLX suppressed atrial fibrillation (AF) in spontaneously hypertensive (12) and aged (13) rats by increasing conduction velocity and reversing fibrosis and cellular hypertrophy. Given these promising data, we now examine the ability of RLX to ameliorate the deleterious cardiac effects of SuHx-induced PAH on the heart.

## Methods

Male Sprague-Dawley rats (300 g, *n* = 30) received the VEGFR2 antagonist, Sugen (SU5416, Sigma) as a subcutaneous injection (20 mg/kg), in 0.5% Na-carboxymethyl cellulose, 0.4% polysorbate, 0.9% NaCl, and 0.9% benzyl alcohol). Rats were implanted with mini-pumps (Alzet Cupertino, CA, Model 2ML4) on day zero to deliver: (i) the vehicle (Na-acetate, *n* = 11), (ii) 30 (*n* = 8) or (iii) 400 μg/kg/day (*n* = 11) of RLX for 6-weeks. The pumps were replaced on day 28. On day zero, the rats were placed in a hypoxic chamber (10% O_2_) for 3-weeks, followed by 3-weeks in normoxia (21% O_2_). Blood samples were taken at the beginning and the end of the experiments and tested for [RLX] (R&D Systems, DRL200-Human Relaxin-2). Relaxin was not detectable at day zero and was 0, 2.6 ± 1 ng/mL and 15 ± 12 ng/mL, respectively, for controls and rats treated with low or high-dose RLX. A fourth group of control rats (*n* = 9) received a subcutaneous injection of vehicle (2 ml/kg) without sugen.

Protocols were approved by the Institutional Animal Care and Use Committee of the University of Pittsburgh and complied with the Guide for the Care and Use of Laboratory Animals (NIH-publication 85-23, revised 1985). Relaxin was the generous gift of Dr. Daniele Bani from the Foundation for Research on Relaxin.

RV hemodynamics were assessed from Doppler flow (DSPW, Indus Instruments, Webster, TX) and RV and LV pressure-volume measurements (Transonic, Scisence and IOX2, EMKA). Briefly, rats were anesthetized (isoflurane), intubated, and the thorax was opened to insert the admittance pressure-volume catheter tip into the RV to measure pressure. Pulmonary artery (PA) Doppler flow velocities were simultaneously measured as PA and RV pressure. All signals were analyzed using Matlab (R2014a, MathWorks, MA).

PA Doppler flow velocity envelopes were traced and used to calculate mean velocity. Stroke distance was determined from the integral of the velocity envelope and converted to stroke volume assuming a 2.5 mm diameter for the PA (stroke volume = stroke distance x cross sectional area). Cardiac output was calculated as the product of stroke volume and heart rate.

Hearts were excised, perfused on a Langendorff apparatus, placed in a custom-made chamber to reduce motion artifacts, and labeled with a voltage-sensitive dye (RH-237) and a Ca^2+^ indicator (Rhod-2/AM) to measure cardiac action potentials (APs) and Ca^2+^ transients (CaTs), respectively (12, 14). Two CMOS cameras (100 × 100 pixels) were used to measure voltage and calcium, each pixel recording from 150 × 150 μm of epicardium (12, 14). Data were recorded for 4–8 s intervals, at 1K frames/s. Activation and repolarization time-points were measured at each pixel from fluorescence (*F*) signals by calculating (d*F*/d*t*)_max_ and (d^2^*F*/d*t*^2^)_max_, respectively. AP duration (APD) was measured from (d*F*/d*t*)_max_ to 75% recovery to baseline, APD_75_. Mean APD_75_ was calculated by averaging APD_75_ from 10 × 10 pixels from each heart. Local conduction velocity (CV) vectors were calculated for each pixel, and mean CV were calculated mean CV ± standard deviation, as previously described (14–16).

Restitution of APDs and CV was measured by programmed stimulation (pacing the LV or RV at 10–20 S1 pulses at 250 ms cycle length (CL) followed by a premature S2 pulse at progressively shorter intervals, until loss of capture or the initiation of an arrhythmia. APD Restitution Kinetics (RK) curves were plotted as mean APD_75_ (100 pixels on RV or LV) vs. S1-S2 interval. CV RK curves were plotted as mean CV vs. S1-S2 (12, 14). Transient ventricular arrhythmia lasted 5–15 s and self-terminated; sustained ventricular arrhythmia lasted >3 min and could be terminated with a bolus of 1M KCl. RV hypertrophy was calculated from RV weights normalized to tibia length and the Fulton index (RV weight/ LV + septum weight) (12, 13).

Cardiac tissues (*n* > 4/group) were fixed in 2% PFA, equilibrated in 30% sucrose, and flash frozen. Frozen sections (5-μm thick) were stained with Mason's Trichrome (not shown) and Rabbit anit-β-catenin antibody. The right bronchus was ligated, and the right lung was stored at −80°C. β-catenin was analyzed using background subtraction in ImageJ (applied equally to each image using the same background subtraction), followed by an analysis of antibody fluorescence only at intercalated disk (12, 17).

Statistical analysis was performed by ANOVA (GraphPad Prism) followed by Tukey post-test comparisons among groups within each data set. Statistical significance was set at *p* < 0.05.

## Results

### PAH Rats

**Figure 1:**
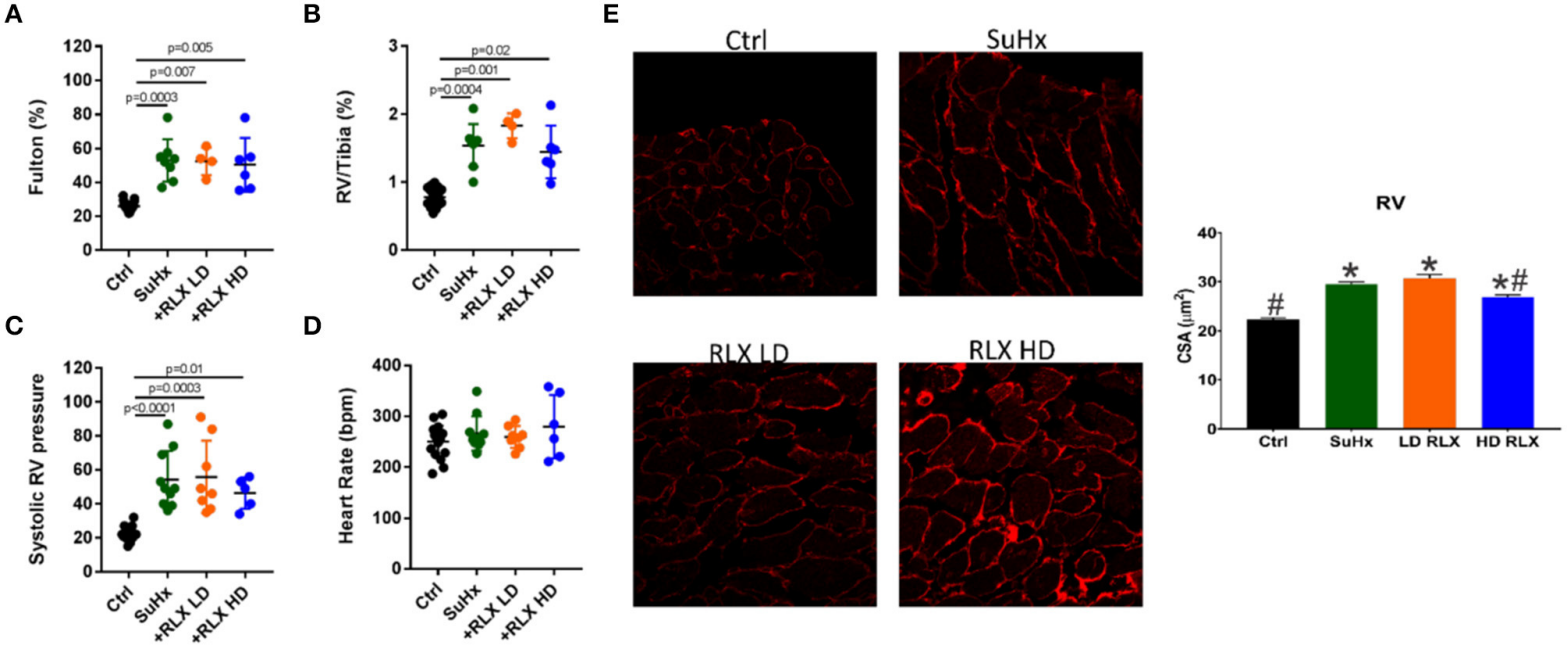
Effects of RLX on RV hypertrophy and pulmonary pressure in PAH. Sugen/Hypoxia significantly increased RV hypertrophy as measured by **(A)** Fulton Index and **(B)** RV/Tibia ratio. Low (+RLX-LD) and high (+RLX-HD) had no effect on RV hypertrophy. **(C)** Sugen/Hypoxia significantly increased systolic pressure and Relaxin failed to alter it. **(D)** Heart rate did not significantly change in all four groups. **(E)** SuHx hearts had a small but significant increase in myocyte cross-sectional area (CSA) (WGA-label) which was prevented by RLX-HD but not RLX-LD; *n* > 100 cells/group. *Indicates *p* < 0.05 vs. control. ^#^Indicates *p* < 0.05 vs. SuHx.

Rats treated with SuHX develop pulmonary arterial hypertension as manifested by significant RV hypertrophy (measured by the Fulton Index andthe RV to Tibia length ratio), an increase in systolic RV pressure, and pulmonary vascular resistance compared to controls (Figures 1A–C). There was no significant change in heart rate between controls and PAH rats (Figure 1D). Neither RLX-LD (Relaxin Low-Dose) or RLX-HD (Relaxin High-Dose) altered RV hypertrophy (Figures 1A,B), systolic RV pressure (Figure 1C) or heart rate (Figure 1D). However, RLX HD prevented cellular hypertrophy by a small but significant reduction in cross-sectional area (CSA) when using wheat germ agglutinin (WGA), an alternative determination of cellular hypertrophy, compared to SuHx rats (Figure 1E). The large number of cells analyzed (>100 cells/group) likely increased the sensitivity to small differences between groups, compared to macroscopic detection of RV hypertrophy (Figures 1A,B).

**Figure 2:**
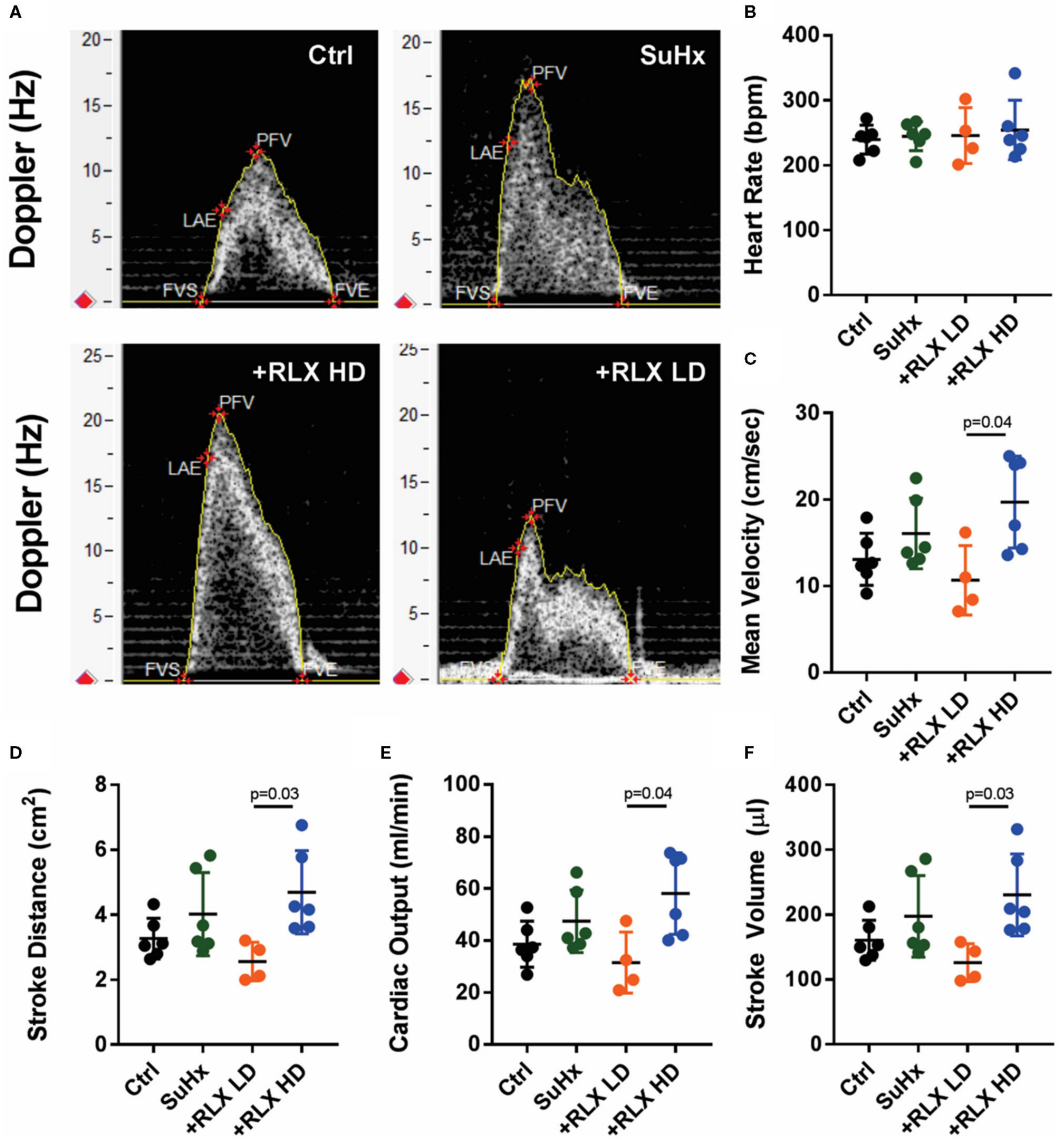
Relaxin significantly altered Doppler flow patterns. **(A)** Doppler flow velocities from the RV outflow-track exhibited marked mid-systolic notching (indicative of decreased pulmonary compliance) and an increase in PFV in SuHx compared to control (Ctrl) rats [**(A)** top panels]. SuHx+ low-dose of RLX (RLX-LD) prevented the increase in PFV but did not alter the notching (bottom right panel). High-dose of RLX (RLX-HD) prevented the notching but not PFV (bottom left panel). PAH (SuHx) rats had no significant changes in **(B)** heart rate, and a tendency to increases in **(C)** mean Velocity, **(D)** Stroke Distance, **(E)** Cardiac Output and **(F)** Stroke Volume compared to controls. RLX-LD had a tendency to prevent the increase in these parameters caused by SuHx **(D–F)** but these reductions did not reach statistical significance. RLX-HD was less effective at preventing the higher mean velocity, stroke distance, cardiac output and stroke volume in SuHx compared to RLX-LD.

Flow velocity profiles in Su-Hx rats showed mid-systolic notching indicative of maladaptive pulmonary vascular remodeling (reduction of pulmonary compliance) and an increase in pulmonary flow velocity (PFV) (Figure 2A, top panels, *n* = 6 hearts/group). RLX-LD prevented the increase in PFV but not the notching profile (Figure 2A, bottom right panel). Notching was markedly reduced and PFV increased in RLX-HD (Figure 2A, bottom left panel). SuHx rats had no significant changes in mean-velocity, stroke distance, cardiac-output, or stroke volume compared to controls (Figures 2B–F). PAH rats treated with RLX HD had a significant increase in mean-velocity, stroke distance, cardiac-output, and stroke volume compared to PAH rats treated with zero or RLX-LD.

**Figure 3:**
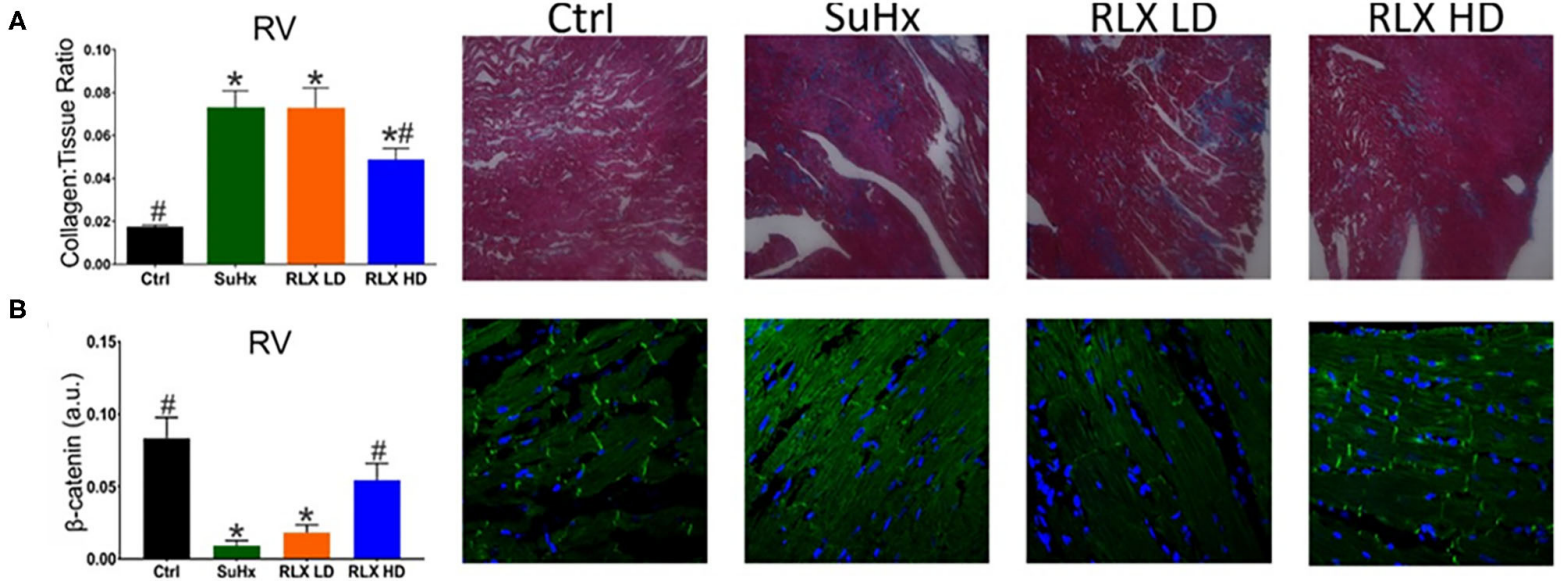
Effects of RLX on RV fibrosis and cell-cell coupling. **(A)** PAH (SuHx) hearts developed an increase in fibrosis that was prevented by treating rats with SuHx + RLX-HD [**(A)**: RLX-HD panel], but not in rats treated with SuHx + RLX-LD [**(A)**: RLX-LD panel]. **(B)** RV cell-cell junctions were disrupted by SuHx treatment compared to controls, as measured by β-catenin expression and β-catenin localization to intercalated disk (Ctrl). PAH rats (SuHx) had a marked reduction of β-catenin expression [**(B)**: SuHx panel] compared to control rats [**(B)**: Ctrl panel]. PAH rats treated with RLX-LD [**(B)**: RLX-LD panel] did not show appreciable differences in β-catenin compared to rats treated with SuHx, alone. In PAH rats, RLX-HD treatment significantly prevented the reduction of β-catenin expression [**(B)**: RLX-HD panel] (*n* ≥ four animals/per group. *Indicates *p* < 0.05 vs. control. ^#^Indicates *p* < 0.05 vs. SuHx.

In PAH, RV remodeling included increased fibrosis (Figure 3A) and a reduction in β-catenin at intercalated-disks (Figure 3B). These maladaptive effects of PAH hearts were suppressed by RLX-HD but not RLX-LD treatment. Immuno-fluorescence was preferentially used over Western blot amnalysis to determine the localization of β-catenin at intercalated disks, since it is an important component of the peptide complex forming the structure of intercalated disks.

### Conduction Velocities (CV) and Restitution Kinetics of CV

**Figure 4:**
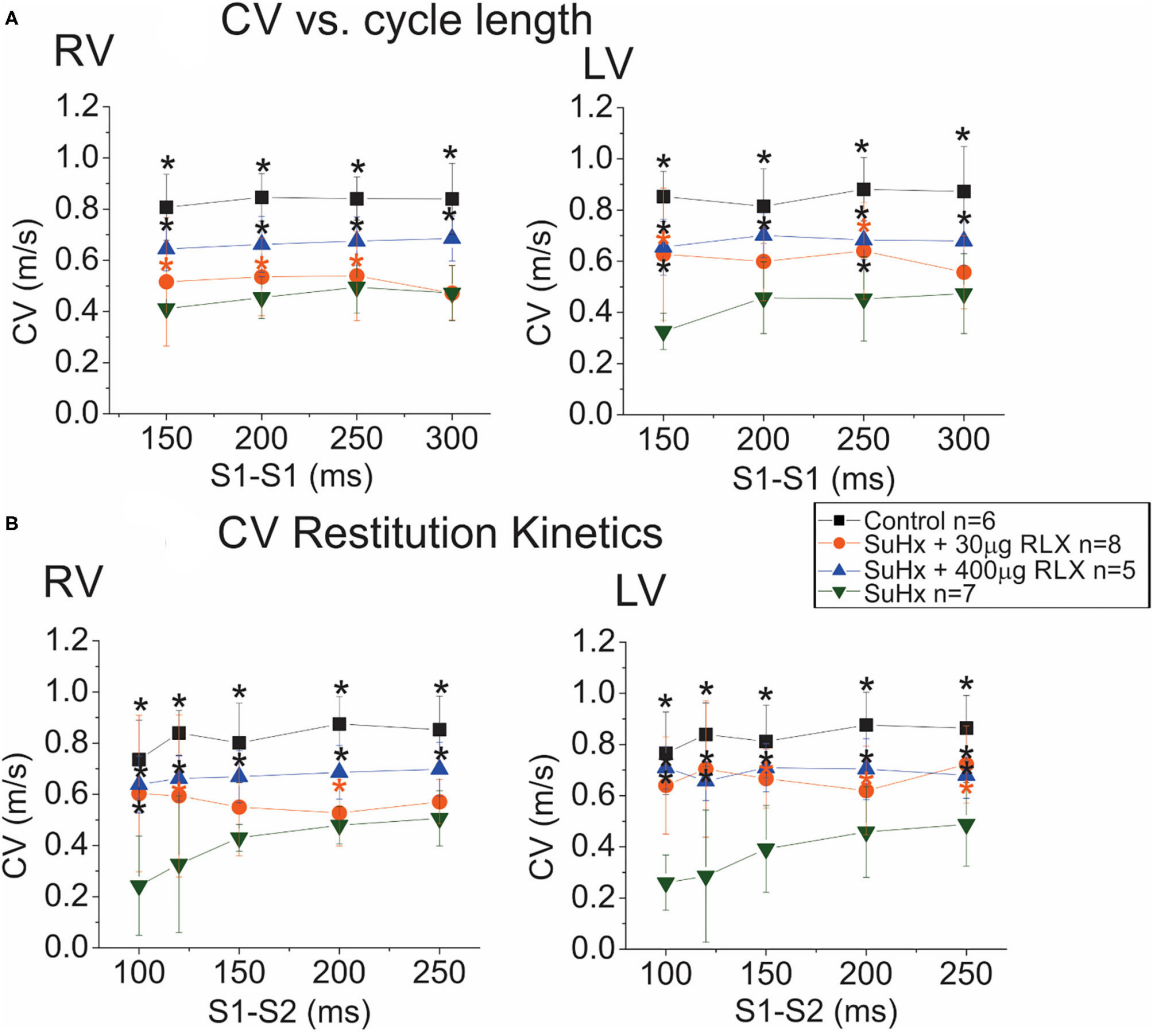
Effects of RLX on CV and Restitution Kinetics (RK). **(A)**: Plots of CV vs. cycle length show a 50% decrease in CV in the RV (left) and LV (right) of SuHx hearts compared to controls. RLX treatments partially prevented CV slowing in a dose-dependent manner. **(B)**: RK plots the CV of the premature AP as a function of S1-S2 inter-pulse interval. CV RK curves of SuHx hearts have a steeper slope and are reduced compared to controls in the RV and LV, and RLX reduces the slope of CV RK curves and increases CVs in a dose-dependent manner. *Indicates *p* < 0.05 vs. SuHx.

The intrinsic heart rate of perfused hearts was monitored under sinus rhythm (SR) followed by programmed stimulation to test the arrhythmia phenotype. All control hearts (*n* = 9) were in SR, and no spontaneous arrhythmias or ectopic beats were noted. Simultaneous recordings of APs and CaTs were initially similar among the four groups (Control, SuHx, SuHx+ RLX-LD, and SuHx + RLX-HD) during SR with no observable dysfunction in CaTs (not shown). Relaxin is known to suppress atrial fibrillation by increasing CV in the atria (12, 13). Here, CV was measured from the epicardium of the RV and LV by pacing each ventricle separately at 300 ms CL. The longitudinal CV of control hearts was 0.84 ± 0.2 m/s in the RV and LV (*n* ≥ 4/group) and was reduced in PAH hearts by 2.2-fold (RV: 0.39 ± 0.14 m/s, LV: 0.38 ± 0.11 m/s; *n* ≥ 4/group) (Figure 4A). RLX-LD partially prevented the effects of PAH on CVs (RV: 0.53 ± 0.15 m/s and LV: 0.62 ± 0.12 m/s, *n* ≥ 4/group), and RLX-HD increased RV and LV CVs by 1.9 and 2.0-fold (*n* ≥ 4/group). RLX-HD hearts had CVs that were not statistically different from controls (RV: 0.79 ± 0.21 m/s, LV: 0.73 ± 0.08 m/s). CVs in the RVs of the RLX LD group were slower than in the RV of the RLX HD group (*p* = 0.02), whereas there was a trend toward slower CVs in the LVs (*p* = 0.09). These data are consistent with previous findings in atria and ventricles (12, 13, 19) and show that RLX increases CVs in a dose-dependent manner.

Restitution kinetics (RK) of CV was measured by applying 20 paced impulses (S1) at 300 ms intervals, followed by a single premature impulse (S2) at variable S1-S2 delays (Figure 4B). RLX-HD, and to a lesser extent, RLX-LD, significantly improved RK of CV relative to SuHx (Figure 4B). Thus, RLX prevented the suppression of CV and RK of CV seen in PAH hearts in a dose-dependent manner. SuHx prolonged AP durations (APDs) measured at APD_75_ and RLX-HD prevented APD prolongation (not shown), but these tendencies did not reach statistical significance.

### Arrhythmia Vulnerability

**Figure 5:**
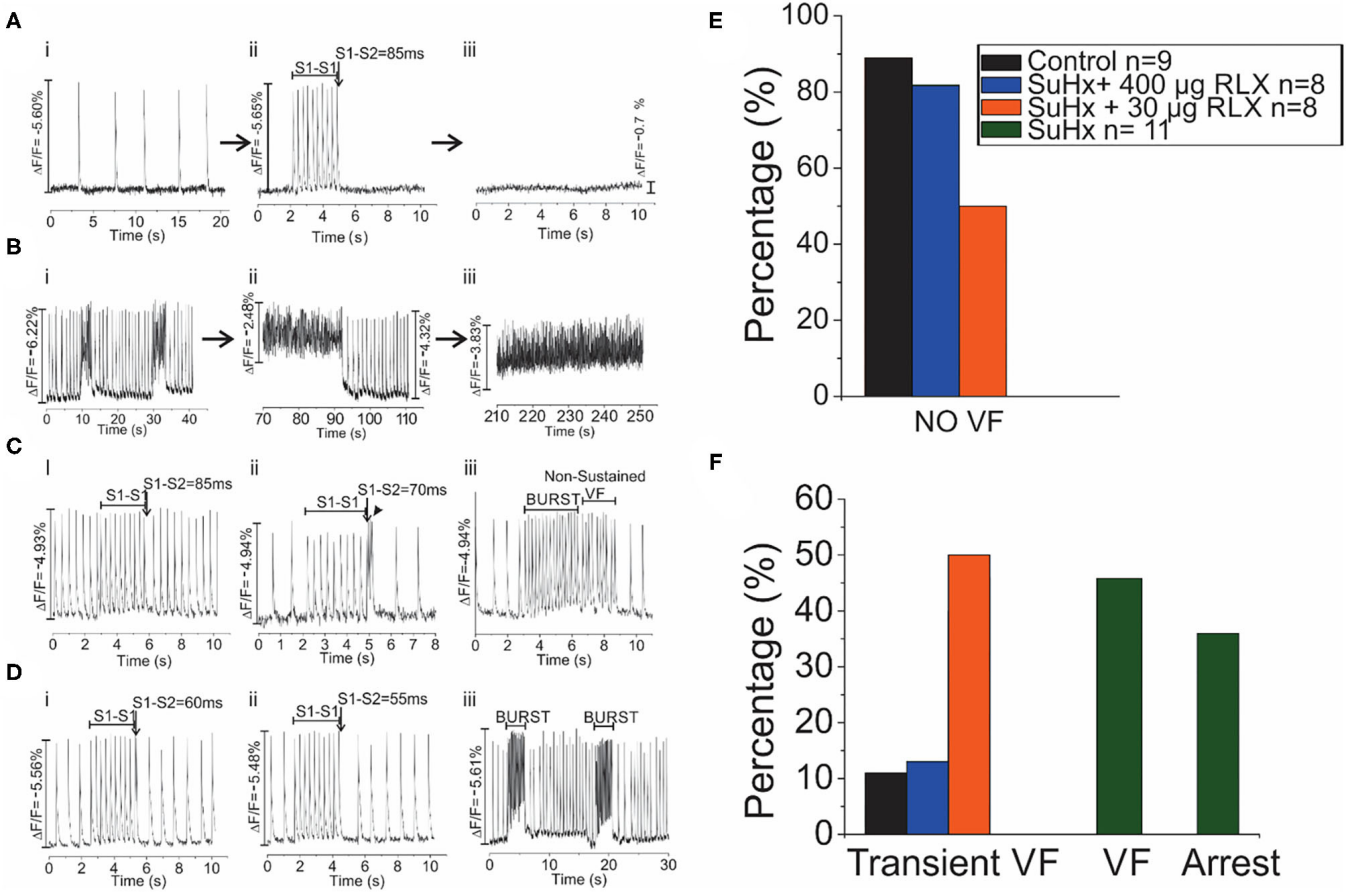
Arrhythmia Phenotypes of PAH hearts. Optical mapping revealed that SuHx hearts exhibited two equally prevalent phenotypes **(A,B)**. **(A)**: SuHx hearts were in bradycardia (i) and could be briefly paced at higher rates (ii) which was followed by cardiac arrest (iii) (*n* = 4/11). Once in cardiac arrest, electrical stimuli (10X threshold, 1–10 ms) failed to capture. **(B)**: Other SuHx hearts exhibited spontaneous ventricular fibrillations (VF) (i) which increased in durations, were self-terminating (ii) and transitioned to a sustained VF, in this illustration in ~ 3 min) (iii) (*n* = 5/11). **(C)**: In the SuHX+RLX LD (RLX LD) group, Relaxin at low dose suppressed both sustained VF and cardiac arrest (*n* = 0/8): (i) AP recordings in sinus rhythm, followed by 2 s of rapid pacing and a premature impulse at S1-S2 of 85 ms which failed to elicit a VF. (ii) In another SuHx+RLX-LD heart, AP record a bradycardia, followed by rapid pacing and a premature impulse (S1-S2 = 70 ms). Here, the premature impulse elicits a brief transient arrhythmia (4 beats) and a return to sinus rhythm. (iii) An example of burst pacing (4 s) which elicits a transient VF that self terminates in <3 s. **(D)**: In the SuHX+RLX HD (RLX HD), Relaxin suppressed cardiac arrest and VF, spontaneous or after burst pacing. (i–ii) examples of rapid pacing and premature impulses of S1-S2 = 60 or 55 ms that failed to elicit VF. (iii) RLX-HD suppressed VF even after repeated burst-pacing events. **(E)**: Summary of VF events after rapid pacing, programmed stimulation and burst pacing. **(F)**: Summary of the 4 groups of hearts that exhibited transient and sustained VF or cardiac arrest.

Control, SuHx, and SuHx+RLX treated hearts underwent programmed stimulation to evaluate their arrhythmia vulnerability. In control hearts, pro-arrhythmic pacing (premature impulse or burst pacing) induced a VF in only 1 out of 9 hearts. In PAH hearts (*n* = 11), two types of electrical dysfunctions were observed. (1) SuHx hearts (*n* = 4/11) had severe bradycardia relative to controls (23 ± 31 beats per minute vs. 137 ± 48, *p* = 0.001) (Figure 5A, i). These hearts could be paced at faster rates for short intervals (5–10 s) then became asystolic. Electrical stimulation of hearts in asystole failed to capture or elicit APs and CaTs (Aii-iii). (2) SuHx hearts had normal heart rates and spontaneous episodes of VF of various durations (2–50 s) (*n* = 5/11) that progressed to sustained VF (*n* = 3/11) (Figure 5B).

**Figure 6:**
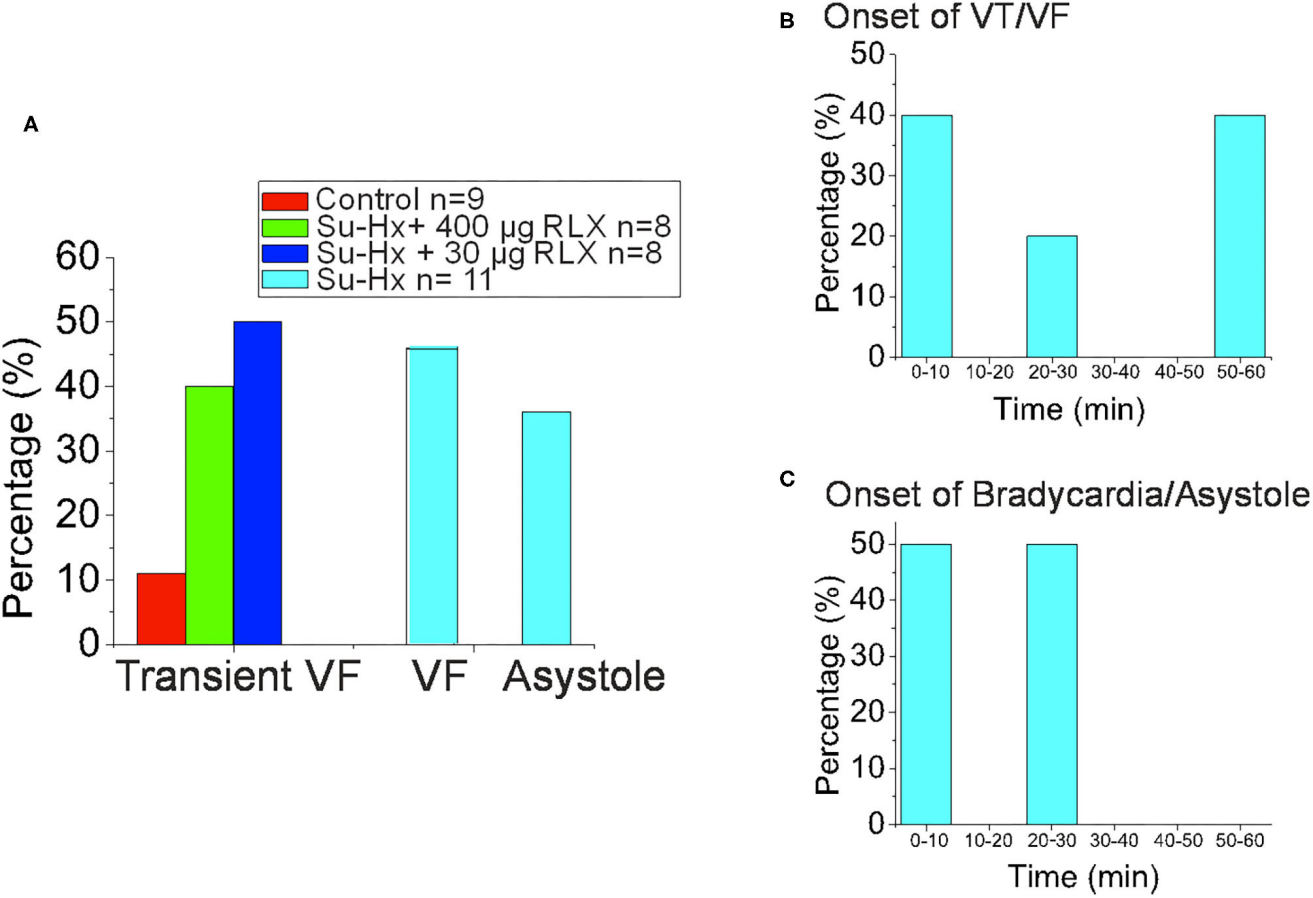
Summary of Experimental Arrhythmia Phenotypes. **(A)** Histogram of the onset of VT/VF. **(B)** Histogram of the Percentage of Hearts that exhibited Transient VF lasting > 10 s, sustained VF or sustained Asystole. **(C)** Histogram of the onset of Bradycardia/Asystole.

Treatment of SuHx PAH rats with RLX-LD (*n* = 8) prevented both types of electrical dysfunctions. Programmed stimulation with premature impulses at S1-S2 = 85 and 70 ms (Figure 5C, i, ii) did not elicit arrhythmias, and burst pacing elicited a few beats of transient VF (>10 s; *n* = 4/8) before recovering to sinus rhythm (Figure 5C, iii). In rats treated with RLX-HD (*n* = 8), programmed stimulation with S1-S2 = 60 and 55 ms failed to induce an arrhythmia (*n* = 0/8; Figure 5D, i-ii); burst pacing also failed to elicit even a brief arrhythmia (Figure 5D, iii, *n* = 1/8). Thus, RLX-HD rescued PAH hearts from bradycardia and asystole as well as sustained arrhythmias elicited either spontaneously or experimentally (*n* = 0/8). Figures 5E,F summarize the arrhythmia phenotypes. A summary of the electrical phenotypes and the time-frame of their occurrence is shown in Figure 6.

**Figure 7:**
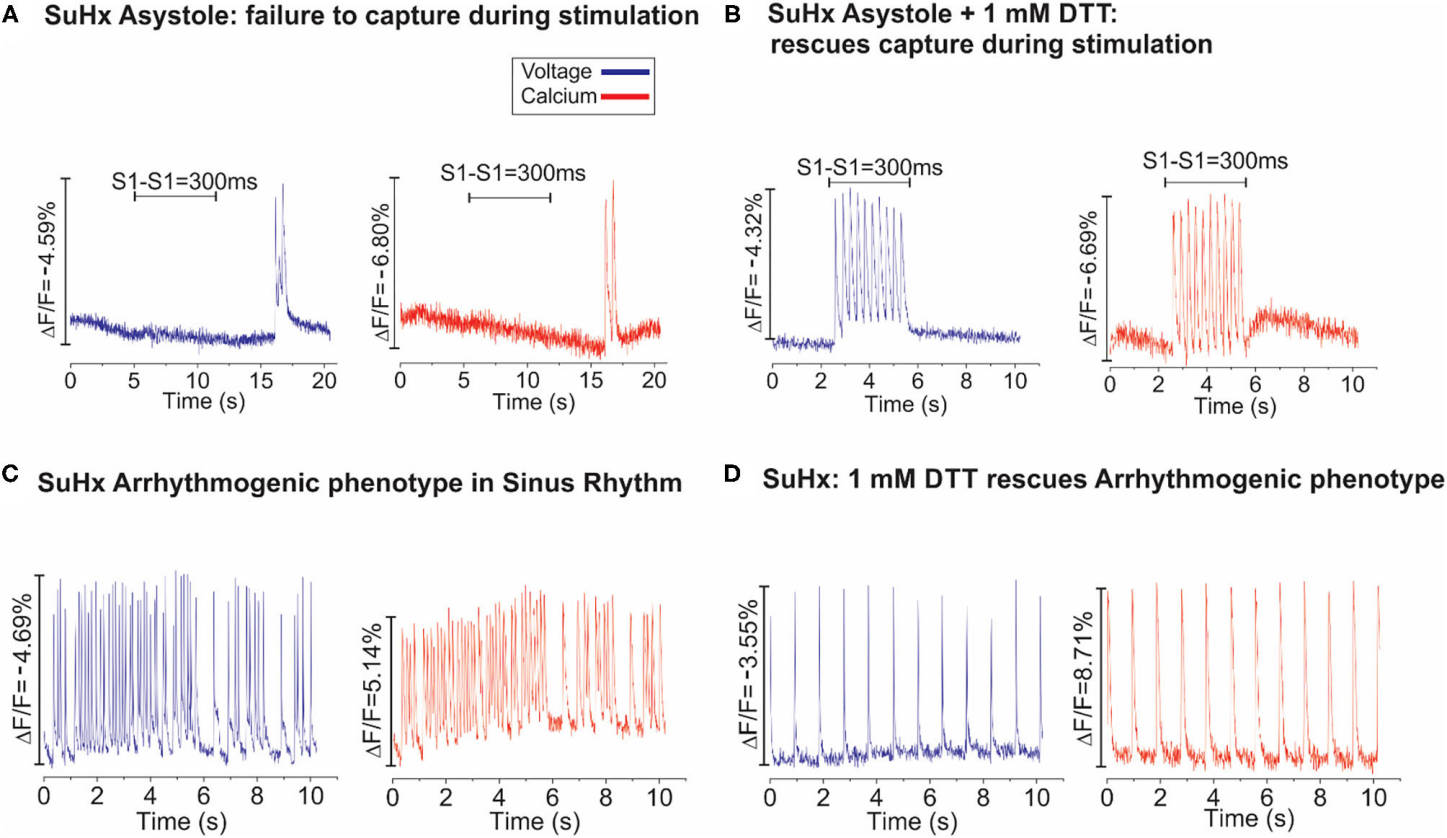
Dithiothreitol (DTT) rescues PAH hearts. **(A)**: Dual optical mapping of voltage (blue) and Ca^2+^ (red) illustrates an asystolic SuHx heart under sinus rhythm that fails to capture during pacing (S1–S1 = 300 ms) with an occasional transient salvo of APs but is otherwise not excitable. **(B)**: The same heart was rescued by perfusing with 1 mM DTT (2–3 min), as electrical impulses now successfully capture at each stimulus (*n* = 2/2 hearts). **(C)**: Voltage and Calcium mapping of a SuHx heart in sinus rhythm illustrates spontaneous bursts of tachycardia which typically progress to sustained VF. **(D)**: Perfusion of the same heart with DTT (1 mM) stabilized the intrinsic sinus rhythm for the remainder of the experiment (*n* = 2/2).

**Figure 8:**
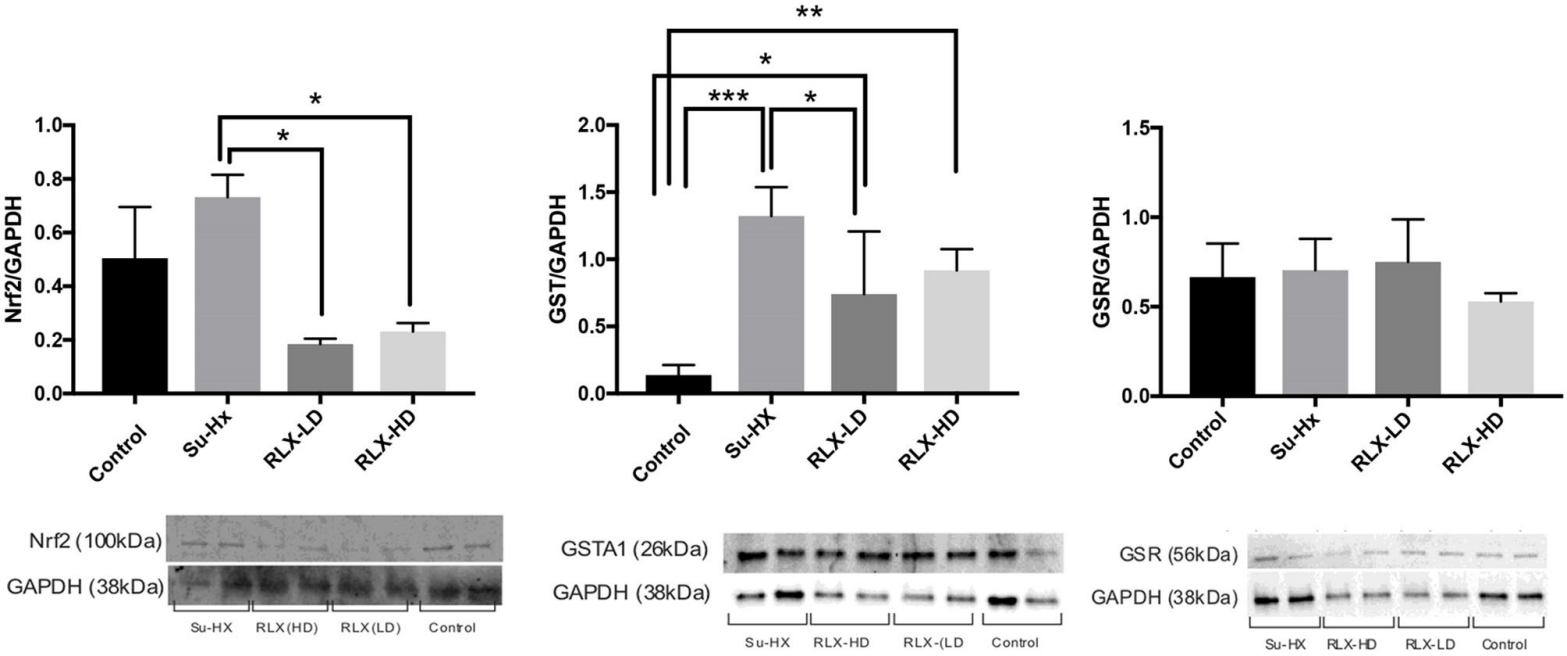
Relaxin prevents oxidative stress in PAH hearts. Relaxin quantitatively prevents the overexpression of Nrf2 and Glutathione Transferase but not Glutathione Reductase. Each lane was loaded with 20 μg of protein and the variability of loaded proteins was due to high protein levels in some tissue samples resulting in the addition of small volumes (2 μl) in some lanes. Data were analyzed by ANOVA followed by Tukey's multiple comparisons test. Only statistically significant differences are noted in the graph (*n* = 4 animals per condition; each sample was run at least in duplicate in separate gels; significance: *p < 0.05; **p < 0.01; ***p < 0.001).

Additional pharmacological and ionic interventions were attempted to rescue PAH hearts. All failed except for an addition of dithiothreitol (1 mM) to the perfusate. Figure 7A illustrates voltage (blue) and calcium (red) signals from a pixel on the RV of a PAH heart. In this example, the heart stopped all spontaneous activity and electrical stimulation failed to elicit APs and CaTs, despite attempting a wide range of pulse duration and amplitude. Here, a rare spontaneous doublet of depolarization occurred, which suggests a potentially rescuable RV (Figure 7A). When the same heart was perfused with DTT (1 mM, 5 min), electrical stimuli (300 ms CL) resulted in the capture and propagation of APs and CaTs (Figure 7B, *n* = 2/2). Another PAH heart, exhibited spontaneous transient VF that typically progressed to sustained VF (Figure 7C) and was rescued by perfusion with DTT (1 mM) resulting in a recovery to sinus rhythm (*n* = 2/2) (Figure 7D). The acute rescue of PAH hearts by DTT indicates a change in thiol REDOX balance, which may have a profound influence on the activation of enzymes, transcriptional regulators, protein kinases, and other signaling molecules such as Nrf2 (18). Consistent with this interpretation, Nrf2 and glutathione transferase (GST) were significantly elevated in PAH ventricles, and RLX-LD and RLX-HD prevented the elevations of Nrf2 and GST but did not change glutathione reductase (GSR) (Figure 8).

## Discussion

PAH initiated through an injury to the pulmonary endothelium with sugen, resulted in a decrease in pulmonary compliance, a significant increase in systolic RV pressure and the expected compensatory remodeling of RV hypertrophy and an increase of cardiac fibrosis. Optical mapping of PAH hearts exposed new cardiac dysfunctions: a marked decrease in conduction velocity (CV), and two striking electrical phenotypes in untreated PAH rats, (a) bradycardia followed by cardiac arrest or (b) spontaneous, self-terminating VF that progressed to sustained VF. RLX-treatment increased CV, improved lung compliance (Doppler flow), and suppressed VF and arrest. The different effects of low and high doses of RLX on Doppler flow and pulmonary compliance suggest RLX signaling via its receptor, Rxfp1 is dose dependent and may differ in depending on the cell type. Dose dependent analysis of RLX on isolated cells would help dissect the mechnisms of action *in situ*. Relaxin prevented the formation of RV fibrosis and increased the expression of β-catenin at the intercalated disk. This is consistent with multiple studies where Relaxin was shown to reverse fibrosis and to increase β-catenin expression (5). The upregulation of β-catenin by RLX concurs with a recent finding showing that RLX activates canonical Wnt signaling, resulting in an increase in Wnt1 and β-catenin and a decrease of Dikkopff protein, with β-catenin localized in the nucleus and co-localized with connexin 43 at intercalated disk (17). Although beyond the scope of this report, the interplay between RLX and Wnt signaling is an exciting topic of further research. Nevertheless, in PAH hearts, RLX prevented VT/VF or cardiac arrest by several mechanisms: (a) prevention of cardiac fibrosis, (b) reduced pulmonary compliance, (c) better cell-cell coupling to achieve a faster CV for action potential propagation, mediated by higher β-catenin expression at intercalated disks, and (d) preventing a thiol oxidative stress, consistent with a reduction of Nrf2 and GST and with the acute actions of DTT.

### Relaxin Is Protective of Thiol Oxidation

There is a large body of studies that link thiol oxidation to cardiac dysfunction at the molecular, cellular and in animal models. The redox state of the cytosolic milieu is in a reduced thiol state in physiological conditions and metabolic energy is required to maintained a reduced milieu. A spectrum of cysteines in various peptides can be oxidized resulting in a loss of enzymatic function [e.g., creatine kinase, SERCA, (19, 20)] or modulation of ion channels [e.g., L-type Ca^2+^ channels (21) and SR Ca^2+^ release channels or ryanodine receptors (RyR) (22)]. The cardiac Ryanodine receptor, RyR2 is particularly sensitive to thiol oxidation and has been shown to contain “critical” or “hyperreactive” thiols which when oxidized and reduced, open and close the channel, respectively (23). Hypereactive thiols are a small subset of low *pKa* thiols (2 out of 100 thiols on RyR2) (23) that are deprotonated at physiological pH and can be oxidized by disulfide formation, S-nitrosation or by binding to heavy metals (22, 24, 25). In sarcoplasmic reticulum (SR) vesicles isolated from ischemic heart muscle, RyR2 oxidation resulted in the opening of the release channel and a reduction of SR Ca^2+^ uptake compared to SR isolated from normoxic myocardium; the subsequent addition of glutathione or DTT to ischemic SR, reduced RyR2, closed the release channel and recovered normal SR Ca^2+^ load (26). The oxidation-reduction of reactive thiols on ryanodine receptors was shown to reversibly open-close the channel (23), which was shown to become oxidized in ischemic (26) and failing hearts (27). A lower ratio of reduced to oxidized glutathione (GSH/GSSG) or the loss of cytosolic GSH leads to abnormal Ca^2+^ handling and Ca^2+^ alternans in a canine model of sudden cardiac death (28). Glutathione dysregulation has been implicated in the etiology and progression of a wide range of human cardiac diseases, including arrhythmia and heart failure (28, 29). There is mounting evidence of energy deficiency in heart failure associated with the disruption of oxidative phosphorylation (30) and augmentation of reactive oxygen species that readily oxidize free thiols (31). This suggests that restoring the redox status can aid in suppression of VF and cardiac arrest.

These data open exciting avenues of study to more fully understand RLX's effects in the heart to suppress arrhythmia, as the data hint that RLX may prevent the deleterious cardiac phenotypes in PAH through multiple pathways, which may include the restoration of the redox state as evidenced by RLX-mediated reversal of upregulated Nrf2 and GST in PAH hearts. This interpretation is consistent with the GSH up-regulation in H9C2 rat cardiac cells by RLX following hypoxia-reoxygenation (32), and a reduction in oxidative stress by RLX in a swine ischemia-reperfusion model (33, 34). Hence, the rescue of VT/VF and arrest by DTT implied that PAH hearts had compromised glutathione metabolism and REDOX balance, which was prevented by RLX.

## Limitations and Conclusion

RLX-HD was cardioprotective through an increased CV and suppression of VF and cardiac arrest. Doppler flow measurements of PAH hearts showed a pronounced secondary flow velocity peak indicative of significant pulmonary vascular remodeling which was eliminated by RLX-HD but not RLX-LD. The study applied a high dose of RLX based on previous studies in spontaneously hypertensive and aged rats, but the resulting serum concentration of RLX (15 ng/ml) did not reach the previous values (75 ng/ml), suggesting differences in the biological activity of RLX in these models. Higher doses might prove to be more beneficial, particularly in terms of improving pulmonary vascular compliance, and dose-response experiments should be done to determine at which concentrations RLX is most effective. The possibility that human Relaxin might elicit an immune response in rats cannot be excluded, particularly if the treatment involves repeated exposures at high doses. So far, we and others have used human Relxin in mice and rats and no detectable untoward reactions have been reported.

The study focused on male rats, yet considerable sex-differences in PAH patients and SuHx rats has been reported (35, 36). It should be noted that RLX was administered during the development of PAH due to concerns that Doppler flow and RV pressure measurements (needed to confirm PAH) might compromise the survival of PAH rats, and impact the subsequent actions of RLX-treatment administered post-PAH. Therefore, this study shows that RLX prevents the development of some of PAH's deleterious effects. However, future work should study the ability of RLX to reverse the effect of PAH on cardiovascular and pulmonary physiology by treatment with RLX after, not during, PAH development. Still, the effects of RLX on SHR and aged rats have demonstrated that RLX reverses cardiac injuries (12, 13). Future studies should examine sex-differences, dose-dependent and pulsatile delivery of RLX, changes in cardiac glutathione, and the potential effects of RLX in the treatment of PAH.

## Data Availability Statement

The raw data supporting the conclusions of this article will be made available by the authors, without undue reservation.

## Ethics Statement

The animal study was reviewed and approved by The University of Pittsburgh: Institutional Animal Care and Use Committee of the University of Pittsburgh and complied with the Guide for the Care and Use of Laboratory Animals (NIH-publication 85-23, revised 1985).

## Author Contributions

BM performed histology and helped draft the manuscript. RR performed histology. BG performed the optical mapping and implanted the mini-pumps. RV analyzed the Doppler data. BH conceived the experiments. JP performed histology and immune-fluorecence. Y-CL oversaw the PAH model, injecting sugen and the hypoxia-normoxia protocol. JH performed the surgeries, RV pressure, and Doppler measurements. ST analyzed pulmonary compliance. AM was our in-house PAH expert on the sugen hypoxia model. MG helped formulate the protocol, provided intellectual and financial resources. GR performed critical western blots. GS developed the research idea and wrote the manuscript. All authors contributed to the article and approved the submitted version.

## Conflict of Interest

The authors declare that the research was conducted in the absence of any commercial or financial relationships that could be construed as a potential conflict of interest.

## References

[1] Bloodworth NC West JD Merryman WD. Microvessel mechanobiology in pulmonary arterial hypertension: cause and effect. Hypertension. (2015) 65:483–9. 10.1161/HYPERTENSIONAHA.114.04652 25534705 PMC4326545

[2] Rajdev A Garan H Biviano A. Arrhythmias in pulmonary arterial hypertension. Prog Cardiovasc Dis. (2012) 55:180–6. 10.1016/j.pcad.2012.06.002 23009914 PMC3832144

[3] Abe K Toba M Alzoubi A Ito M Fagan KA Cool CD. Formation of plexiform lesions in experimental severe pulmonary arterial hypertension. Circulation. (2010) 121:2747–54. 10.1161/CIRCULATIONAHA.109.927681 20547927

[4] Rafikova O Rafikov R Kumar S Sharma S Aggarwal S Schneider F. Bosentan inhibits oxidative and nitrosative stress and rescues occlusive pulmonary hypertension. Free Radic Biol Med. (2013) 56:28–43. 10.1016/j.freeradbiomed.2012.09.013 23200808 PMC3749888

[5] Martin B Romero G Salama G. Cardioprotective actions of relaxin. Mol Cell Endocrinol. (2019) 487:45–53. 10.1016/j.mce.2018.12.016 30625345

[6] Tozzi CA Poiani GJ McHugh NA Shakarjian MP Grove BH Samuel CS. Recombinant human relaxin reduces hypoxic pulmonary hypertension in the rat. Pulm Pharmacol Ther. (2005) 18:346–53. 10.1016/j.pupt.2005.01.003 15939313

[7] Unemori EN Pickford LB Salles AL Piercy CE Grove BH Erikson ME. Relaxin induces an extracellular matrix-degrading phenotype in human lung fibroblasts *in vitro* and inhibits lung fibrosis in a murine model *in vivo*. J Clin Invest. (1996) 98:2739–45. 10.1172/JCI119099 8981919 PMC507738

[8] Samuel CS Zhao C Bathgate RA Bond CP Burton MD Parry LJ. Relaxin deficiency in mice is associated with an age-related progression of pulmonary fibrosis. FASEB J. (2003) 17:121–3. 10.1096/fj.02-0449fje 12424226

[9] Dschietzig T Teichman S Unemori E Wood S Boehmer J Richter C. Intravenous recombinant human relaxin in compensated heart failure: a safety, tolerability, and pharmacodynamic trial. J Card Fail. (2009) 15:182–90. 10.1016/j.cardfail.2009.01.008 19327619

[10] Teichman SL Unemori E Dschietzig T Conrad K Voors AA Teerlink JR. Relaxin, a pleiotropic vasodilator for the treatment of heart failure. Heart Fail Rev. (2009) 14:321–9. 10.1007/s10741-008-9129-3 19101795 PMC2772950

[11] Teichman SL Unemori E Teerlink JR Cotter G Metra M. Relaxin: review of biology and potential role in treating heart failure. Curr Heart Fail Rep. (2010) 7:75–82. 10.1007/s11897-010-0010-z 20424993 PMC2875472

[12] Parikh A Patel D McTiernan CF Xiang W Haney J Yang L. Relaxin suppresses atrial fibrillation by reversing fibrosis and myocyte hypertrophy and increasing conduction velocity and sodium current in spontaneously hypertensive rat hearts. Circ Res. (2013) 113:313–21. 10.1161/CIRCRESAHA.113.301646 23748429 PMC3774019

[13] Henry BL Gabris B Li Q Martin B Giannini M Parikh A. Relaxin suppresses atrial fibrillation in aged rats by reversing fibrosis and upregulating Na+ channels. Heart Rhythm. (2016) 13:983–91. 10.1016/j.hrthm.2015.12.030 26711798 PMC4801709

[14] Salama G Hwang SM. Simultaneous optical mapping of intracellular free calcium and action potentials from Langendorff perfused hearts. Curr Protoc Cytom. (2009) 49:12.17.1–31. 10.1002/0471142956.cy1217s49 19575468 PMC4536850

[15] Choi BR Nho W Liu T Salama G. Life span of ventricular fibrillation frequencies. Circ Res. (2002) 91:339–45. 10.1161/01.RES.0000031801.84308.F4 12193467

[16] Efimov IR Huang DT Rendt JM Salama G. Optical mapping of repolarization and refractoriness from intact hearts. Circulation. (1994) 90:1469–80. 10.1161/01.CIR.90.3.1469 8087954

[17] Martin B Gabris B Barakat AF Henry BL Giannini M Reddy RP. Relaxin reverses maladaptive remodeling of the aged heart through Wnt-signaling. Sci Rep. (2019) 9:18545. 10.1038/s41598-019-53867-y 31811156 PMC6897890

[18] Sies H Jones DP. Reactive oxygen species (ROS) as pleiotropic physiological signalling agents. Nat Rev Mol Cell Biol. (2020) 21:363–83. 10.1038/s41580-020-0230-3 32231263

[19] Rech VC Mezzomo NJ Athaydes GA Feksa LR Figueiredo VC Kessler A. Thiol/disulfide status regulates the activity of thiol-containing kinases related to energy homeostasis in rat kidney. An Acad Bras Cienc. (2018) 90:99–108. 10.1590/0001-3765201720160348 29236866

[20] Vazquez P Tirado-Cortes A Alvarez R Ronjat M Amaya A Ortega A. Reversible oxidation of vicinal-thiols motif in sarcoplasmic reticulum calcium regulatory proteins is involved in muscle fatigue mechanism. Cell Calcium. (2016) 60:245–55. 10.1016/j.ceca.2016.06.001 27422341

[21] Hool LC. Evidence for the regulation of l-type Ca2+ channels in the heart by reactive oxygen species: mechanism for mediating pathology. Clin Exp Pharmacol Physiol. (2008) 35:229–34. 10.1111/j.1440-1681.2007.04727.x 18197892

[22] Salama G Abramson J. Silver ions trigger Ca2+ release by acting at the apparent physiological release site in sarcoplasmic reticulum. J Biol Chem. (1984) 259:13363–9. 10.1016/S0021-9258(18)90703-9 6208194

[23] Zaidi NF Lagenaur CF Abramson JJ Pessah I Salama G. Reactive disulfides trigger Ca2+ release from sarcoplasmic reticulum via an oxidation reaction. J Biol Chem. (1989) 264:21725–36. 10.1016/S0021-9258(20)88246-5 2532212

[24] Abramson JJ Salama G. Critical sulfhydryls regulate calcium release from sarcoplasmic reticulum. J Bioenerg Biomembr. (1989) 21:283–94. 10.1007/BF00812073 2666411

[25] Salama G Menshikova EV Abramson JJ. Molecular interaction between nitric oxide and ryanodine receptors of skeletal and cardiac sarcoplasmic reticulum. Antioxid Redox Signal. (2000) 2:5–16. 10.1089/ars.2000.2.1-5 11232600

[26] Menshikova EV Salama G. Cardiac ischemia oxidizes regulatory thiols on ryanodine receptors: captopril acts as a reducing agent to improve Ca2+ uptake by ischemic sarcoplasmic reticulum. J Cardiovasc Pharmacol. (2000) 36:656–68. 10.1097/00005344-200011000-00016 11065227

[27] Terentyev D Gyorke I Belevych AE Terentyeva R Sridhar A Nishijima Y. Redox modification of ryanodine receptors contributes to sarcoplasmic reticulum Ca2+ leak in chronic heart failure. Circ Res. (2008) 103:1466–72. 10.1161/CIRCRESAHA.108.184457 19008475 PMC3274754

[28] Belevych AE Terentyev D Viatchenko-Karpinski S Terentyeva R Sridhar A Nishijima Y. Redox modification of ryanodine receptors underlies calcium alternans in a canine model of sudden cardiac death. Cardiovasc Res. (2009) 84:387–95. 10.1093/cvr/cvp246 19617226 PMC2777950

[29] Ballatori N Krance SM Notenboom S Shi S Tieu K Hammond CL. Glutathione dysregulation and the etiology and progression of human diseases. Biol Chem. (2009) 390:191–214. 10.1515/BC.2009.033 19166318 PMC2756154

[30] Sheeran FL Pepe S. Energy deficiency in the failing heart: linking increased reactive oxygen species and disruption of oxidative phosphorylation rate. Biochim Biophys Acta. (2006) 1757:543–52. 10.1016/j.bbabio.2006.03.008 16631107

[31] Koning AM Meijers WC Pasch A Leuvenink HGD Frenay AS Dekker MM. Serum free thiols in chronic heart failure. Pharmacol Res. (2016) 111:452–8. 10.1016/j.phrs.2016.06.027 27378569

[32] Nistri S Fiorillo C Becatti M Bani D. Human relaxin-2 (serelaxin) attenuates oxidative stress in cardiac muscle cells exposed *in vitro* to hypoxia-Reoxygenation. Evidence for the involvement of reduced glutathione up-regulation. Antioxidants. (2020) 9:774. 10.3390/antiox9090774 32825567 PMC7555919

[33] Zhao Z Ng CY Liu T Li H Li G. Relaxin as novel strategy in the management of atrial fibrillation: potential roles and future perspectives. Int J Cardiol. (2014) 171:e72–3. 10.1016/j.ijcard.2013.11.103 24373631

[34] Perna AM Masini E Nistri S Briganti V Chiappini L Stefano P. Novel drug development opportunity for relaxin in acute myocardial infarction: evidences from a swine model. FASEB J. (2005) 19:1525–7. 10.1096/fj.04-3664fje 16009702

[35] Tofovic SP Bilan VP Jackson EK Schneider F. Sugene 5416 dose-hypoxia-normoxia-gender interaction in angioproliferative pulmonary hypertension in rats. Am J Respir Crit Care Med. (2014) 189:A5566. 10.1164/ajrccm-conference.2014.189.1_MeetingAbstracts.A5566

[36] Stacher E Graham BB Hunt JM Gandjeva A Groshong SD McLaughlin VV. Modern age pathology of pulmonary arterial hypertension. Am J Respir Crit Care Med. (2012) 186:261–72. 10.1164/rccm.201201-0164OC 22679007 PMC3886716

